# Teachers’ acculturation in culturally diverse schools - How is the perceived diversity climate linked to intercultural self-efficacy?

**DOI:** 10.3389/fpsyg.2022.953068

**Published:** 2022-10-21

**Authors:** Jolina Ulbricht, Maja K. Schachner, Sauro Civitillo, Peter Noack

**Affiliations:** ^1^Department of Educational Psychology, Martin-Luther-University Halle-Wittenberg, Halle, Germany; ^2^Department of Geography Didactics, Martin-Luther-University Halle-Wittenberg, Halle, Germany; ^3^Department of Interdisciplinary Social Science, Utrecht University, Utrecht, Netherlands; ^4^Department of Educational Psychology, University of Jena, Jena, Germany

**Keywords:** school climate, acculturation attitudes, acculturation, teacher, self-efficacy

## Abstract

While in the school context, acculturation is often studied in relation to students of immigrant descent, the current study applies an acculturation framework to teachers mostly representing the mainstream culture. Specifically, we investigated whether teachers’ acculturation attitudes towards their students mediate effects of the perceived cultural diversity climate at school on teachers’ intercultural self-efficacy in culturally diverse classrooms. Analyses were based on reports of 186 teachers (14% of immigrant descent; *M*_age_ = 40.8; *SD*_age_ = 11.8, 73% female) in 22 culturally diverse secondary schools in Southwest Germany. Path analyses indicated that perceived norms of *cultural pluralism*, and perceived norms of *equality and inclusion* are directly and positively associated with facets of intercultural self-efficacy. Moreover, teachers’ support for cultural maintenance amongst their students was associated with intercultural self- efficacy, but no mediation was found between climate and intercultural self-efficacy *via* acculturation attitudes. Implications for teacher training, educational practice and future research on the acculturation and adjustment of teachers in culturally diverse classrooms are discussed.

## Introduction

Most societies are characterized by a high cultural diversity, which often results from migration movements. The proportion of international migrants globally increased to an estimated 272 million in 2019 (United Nations, 2019). With global issues such as climate change, war, and increasing social and economic disparities this rising trend will continue. The steady rise in migration is also increasing cultural and ethnic diversity^1^ in classrooms. According to the Mikrozensus (2020) more than one-third (38%) of students at general and vocational schools in Germany are of immigrant descent, meaning that they are immigrants themselves or the children of immigrants. In stark contrast, only 7.2% of the elementary and secondary school teaching staff in Germany are of immigrant descent (Mikrozensus, 2015).

Concerning increasingly diverse schools, teachers are considered agents of change, as they can contribute to a school climate that recognizes and addresses the needs of students of immigrant descent (Brown et al., 2021). Moreover, diversity-related aspects of the school climate are an important condition for acculturation at school (Schachner et al., 2016). Acculturation is defined as the psychological consequences of regular intercultural contact (Berry, 2003). Although acculturation has been researched extensively in recent decades, the focus has mainly been on individuals of immigrant descent, whereas little attention has been paid to psychological changes taking place amongst members of the mainstream society, especially concerning their acculturation attitudes and effects of those on their own functioning in a multicultural society (Haugen and Kunst, 2017; Schachner, 2019). This is particularly dramatic as the lack of research on acculturation of members of the mainstream culture has led to the common misconception that only people of immigrant descent undergo cultural change (Dinh and Bond, 2008; Kunst et al., 2021).

In this study, we apply an acculturation framework to teachers mostly representing the mainstream culture. The framework distinguishes between different components of the acculturation process: The school context can be conceptualized as an acculturation condition that shapes the acculturation experience (Arends-Tóth and van de Vijver, 2006; Schachner et al., 2014, 2018a). We focus on two facets of cultural diversity climate, namely *equality and inclusion*, and *cultural pluralism*, which have been shown to be relevant for the acculturation of students of immigrant descent and teacher outcomes (Schachner et al., 2016; Civitillo et al., 2017). Acculturation attitudes, comprising own acculturation orientations and expectations towards others, constitute the center of the acculturation framework and mediate between acculturation conditions and outcomes (Arends-Tóth and van de Vijver, 2006).

To adequately meet the needs of culturally diverse classrooms, teachers need to be prepared (Gay, 2010; OECD, 2010; Barrett, 2018; Civitillo and Juang, 2019). In this context, intercultural competence is a key qualification which is defined as the “ability to develop targeted knowledge, skills and attitudes that lead to visible behavior and communication that are both effective and appropriate in intercultural interactions” (Deardorff, 2006; p. 247). A main component related to teachers’ intercultural competence is their sense of self-efficacy, as high levels of self-efficacy have positive effects on multiple student and teacher outcomes (for a recent review, see Romijn et al., 2020). To date, little attention has been paid to teacher self-efficacy as an outcome of the acculturation process and how (contextual) acculturation conditions and attitudes relate to this efficacy (Geerlings et al., 2018).

**Figure 1:**
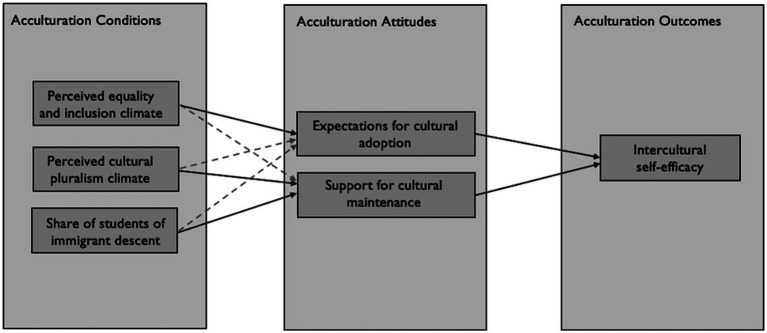
Conceptual model.

Prior research on students has already indicated that the positive effects of a cultural diversity climate (*equality and inclusion*; *cultural pluralism*) on achievement and school adjustment are mediated by students’ own acculturation orientations (Schachner et al., 2016). Based on these findings, our study is the first to apply an acculturation framework to teachers and hypothesize that teachers’ acculturation attitudes (in the sense of their expectations towards the acculturation of their students) mediate the association between the perceived cultural diversity climate in their school and their intercultural self-efficacy (Figure 1). In the following sections, we first introduce the role of the diversity climate as an acculturation condition in the school context. Then, we lay out the significance of teachers’ acculturation attitudes in the school context. Finally, we review the conditions for the development of teachers’ intercultural self-efficacy.

### Cultural diversity climate: *Equality and inclusion* and *cultural pluralism*

Drawing on research on the psychology of intergroup relations (Allport et al., 1954; Tajfel and Turner, 1986) as well as research on a multicultural ideology (Berry, 1977, 2017), two main approaches of dealing with cultural diversity have been distinguished by social psychologists as describing the norms in a particular social context (Park and Judd, 2005; Rosenthal and Levy, 2010; Rattan and Ambady, 2013; Guimond et al., 2014): the first one focuses on overcoming group boundaries (through positive contact) and promoting equal treatment, often termed an equality or color-blind approach, while the second explicitly values diversity, difference and pluralism, often termed a multicultural approach. These approaches often co-occur and have also been found suitable to describe how schools deal with cultural diversity. They are visible at different levels in the school context, such as in school policies (Celeste et al., 2019), student and teacher perceptions of the cultural diversity climate (Schachner et al., 2016, 2021; Civitillo et al., 2018), as well as teacher’s diversity beliefs (Hachfeld et al., 2015; Civitillo et al., 2021).

In conceptualizations of the classroom cultural diversity climate, an equality or color-blind approach implying the promotion of positive contact, equal treatment and cooperation is subsumed under broad concepts such as *interpersonal interactions* (Byrd, 2015, 2017) or *equality and inclusion* (Schachner et al., 2016; Schachner, 2019). A climate fostering *equality and inclusion* in schools aims to promote positive contact between culturally diverse students to overcome differences and prevent stereotyping. Teachers have a crucial role in promoting *equality and inclusion* by acting as role models for equal treatment of all students and actively promoting positive intergroup attitudes (Schwarzenthal et al., 2018). Teachers can foster *equality and inclusion* by encouraging collaboration on common goals through cooperative learning techniques (Banks, 2015; Schachner et al., 2016, 2021; Civitillo et al., 2017). The approach is associated with lower prejudice and discrimination, and generally more positive intergroup experiences amongst students (Schwarzenthal et al., 2018; Schachner et al., 2021).

When perceiving a stronger endorsement of norms of *equality and inclusion*, students of immigrant descent also expressed a stronger orientation towards the mainstream culture (Schachner et al., 2016). However, a strong emphasis on *equality and inclusion* may sometimes imply ignoring or downplaying differences between cultural groups as in colorblindness, which has been associated with a lower awareness of cultural diversity amongst teachers, and lower culturally responsive teaching self-efficacy (Wang et al., 2014; Cadenas et al., 2021).

On the other hand, *cultural pluralism* as a second facet in conceptualizations of the classroom cultural diversity climate values cultural diversity as enriching all students’ learning experiences and is reflected in policies that acknowledge differences in order to value them as positive resources and create a climate that welcomes and appreciates cultural diversity (Schachner et al., 2016; Celeste et al., 2019). Teachers promote norms of *cultural pluralism* in their classrooms by offering many opportunities to engage with diverse cultural norms, traditions, values, and specifically showing an interest in and valuing the cultural backgrounds of the families of their students. When students perceived a stronger support of cultural pluralism, students of immigrant descent held more positive ethnic identities (Brown and Chu, 2012) and were oriented more towards the maintenance of their heritage culture (Schachner et al., 2016). A stronger support of *cultural pluralism* is associated with better teacher-student relationships, with more positive peer interactions, and with an enhanced sense of school belonging (Smith et al., 2020). In summary, both approaches of dealing with diversity in school are mostly associated with positive student and teacher outcomes. Therefore, we hypothesize that a stronger endorsement of *equality and inclusion* as well as *cultural pluralism* is associated with higher intercultural self-efficacy.

### Acculturation in the school context

As schools provide important opportunities for intergroup contact and acculturation amongst children and adolescents (Horenczyk and Tatar, 2012; Motti-Stefanidi et al., 2012; Schachner et al., 2018a,b), they can be viewed as a micro-system, reflecting norms and values within the macro-system of the society at large and influencing individual development (Bronfenbrenner and Morris, 2007). In this process, teachers in culturally diverse schools may also undergo development and experience acculturation (Makarova and Herzog, 2013; Makarova et al., 2019), as well as playing a key role for the development and acculturation of their students in predominantly representing the mainstream cultural group.

Acculturation is defined by a two-dimensional structure: first, maintenance of heritage culture and identity, and second, relationships sought among groups and participation in a common society (Berry, 1997, 2003). However, other researchers have later revised the second dimension by replacing relationships and contact with the mainstream society with the cultural adoption, i.e., adopting key aspects of the mainstream culture (Bourhis et al., 1997). By combining the two dimensions, Berry distinguished four acculturation strategies (Berry, 1997, 2003): integration (high maintenance and high contact), assimilation (low maintenance and high contact), separation (high maintenance and low contact), marginalization (low maintenance and low contact). These strategies and attitudes can be held by both, cultural mainstream society members and people of immigrant descent. Focusing on the cultural mainstream perspective, members of the cultural mainstream society have different expectations about the acculturation of people of immigrant descent, which are defined as *acculturation expectations* (Berry, 2017). Although the four combined patterns are useful conceptually, the statistical classification of acculturation attitudes into four strategies has been criticized for being difficult to replicate and not strictly comparable across studies (van de Vijver, 2017). Empirically we will therefore work with the two dimensions of acculturation expectations as conceptualized by Bourhis et al. (1997), i.e., support for cultural maintenance and expectations for cultural adoption, independently.

Integration was long regarded as the most preferred acculturation strategy for both, people of immigrant descent and members of the cultural mainstream society, as it was associated with the best adjustment outcomes (i.e., higher life-satisfaction, less acculturative stress; Nguyen and Benet-Martínez, 2013; Lefringhausen and Marshall, 2016; Kunst, 2021). However, Germany is a country where the expectations of members of the cultural mainstream society for assimilation are particularly strong and where immigrants’ maintaining elements of their heritage culture is often associated with separation in the public discourse (Zick et al., 2001; Kunst and Sam, 2014).

Teachers may differ in their acculturation attitudes, including acculturation-related expectations they hold towards students (Makarova and Herzog, 2013; Van Praag et al., 2016). Teachers’ acculturation expectations are crucial, as they may serve as particular risk or resource factors for acculturation and school adjustment of students of immigrant descent (Makarova et al., 2019). In addition, teachers’ acculturation expectations are relevant for the well-being of both teachers and students: Teacher with assimilation attitudes run a high risk of stress and stress-related health problems, as well as diversity-related burnout, leaving teachers feeling less able to cope with the challenges of a multicultural classroom (Dubbeld et al., 2019). Moreover, teachers who endorse more assimilative attitudes report lower levels of intercultural self-efficacy, while multicultural attitudes were associated with higher levels of intercultural self-efficacy (Tatar et al., 2011; Hachfeld et al., 2015; Gutentag et al., 2018). A study by Makarova and Herzog (2013) further showed that teachers’ acculturation attitudes are associated with diagnostic competencies in assessing students’ characteristics, social dynamics in the classroom and classroom management. Teachers with integrative attitudes seemed to be best able to prevent social conflict among their students, while teachers with assimilative attitudes showed low diagnostic expertise in the area of social tensions and seemed to be less likely to be successful in preventing social conflicts among their students.

Yet, we know little about how the acculturation expectations of members of the mainstream culture are formed (Acker and Vanbeselaere, 2011), specifically in the context of the cultural diversity climate in schools, and how they may in turn be linked to teachers’ own adjustment in the context of multicultural schools, including their intercultural self-efficacy.

### Teachers’ intercultural self-efficacy in diverse classrooms

According to Bandura’s social cognitive theory (1977), self-efficacy refers to an individual’s judgement of capability to perform a particular action. Teachers’ sense of self-efficacy has been defined as “the teacher’s belief in his or her capability to organize and execute courses of action required to successfully accomplish a specific teaching task in a particular context” (Tschannen-Moran et al., 1998, p. 22).

The relationship between teachers’ self-efficacy and practice is described as reciprocal, and self-efficacy beliefs can work as self-fulfilling prophecies (Tschannen-Moran and Hoy, 2007). Teachers’ perceiving their teaching performance as being successful raises their self-efficacy beliefs and contributes to their expectations of future success. Teachers’ self-efficacy is considered one of the most important beliefs influencing teachers’ professional behaviors (Durksen et al., 2017) and supporting positive and effective classroom behaviors (Zee and Koomen, 2016). In addition, teachers’ self-efficacy has been associated with valuable educational outcomes for both students and teachers. For instance, more self-efficacious teachers are less likely to experience burnout and diversity-related stress and are more satisfied in their profession (Vieluf et al., 2013; Gutentag et al., 2018). Moreover, students of self-efficacious teachers demonstrate greater motivation, academic adjustment, and achievement (Fernet et al., 2012; Wang et al., 2015; Lazarides and Warner, 2020).

Teachers’ intercultural self-efficacy specifically has been conceptualized based on different theoretical concepts, such as multicultural teacher education (Banks and Banks, 2004) and culturally responsive teaching (Gay, 2002). According to these concepts, teachers with high intercultural self-efficacy beliefs are confident to use students’ diverse cultural backgrounds as a learning resource to build positive relationships with students, adapt instruction to diverse students, and create an inclusive learning environment. However, teachers do not always feel prepared in culturally diverse classrooms (Glock et al., 2019a; Slot et al., 2019), as managing the academic, behavioral, communication, and social needs of students can be challenging for teachers (Farmer et al., 2019).

Previous research showed associations between facets of the school climate and teacher self-efficacy (Pas et al., 2012; Aldridge and Fraser, 2016; Schwarzenthal et al., 2022). A cultural diversity climate reflecting *equality and inclusion* and *cultural pluralism* was found to be an important condition for positive intercultural relations amongst students (Schachner et al., 2015; Schwarzenthal et al., 2017). Moreover, a positive cultural diversity climate in schools has been associated with intercultural competence amongst students (Schwarzenthal et al., 2019a). Therefore, we expect similar effects also for the intercultural self-efficacy of teachers. Specifically, we expect that the more teachers perceive their school climate as supporting both *cultural pluralism* and *equality and inclusion*, the higher their intercultural self-efficacy.

To date, little is known about teacher self-efficacy in relation to students from different cultural backgrounds (Geerlings et al., 2018). Teaching an increasingly culturally diverse student population, may imply new demands such as developing an understanding of the contributions of different cultural groups and adapting the curriculum correspondingly.

According to the contact hypothesis (Allport et al., 1954), having more frequent opportunities for intercultural contact can reduce prejudice. Moreover, more intercultural contact is associated with more mutual acceptance and a higher preference for integration (Berry et al., 2022). Therefore, in this study, we control for the proportion of students of immigrant descent as an indicator of opportunity for intergroup contact. Indeed, the proportion of students of immigrant descent is an important characteristic of culturally diverse schools and classrooms and was related to similar outcomes (e.g., teacher attitudes, classroom interactions, acculturation orientations) in previous research (Brault et al., 2014; Schachner et al., 2016; Ortega et al., 2020). Geerlings et al. (2018) also found that Dutch teachers in classes with a higher proportion of students of immigrant descent felt more efficacious in teaching these students compared to teachers in classrooms with a smaller share of students of immigrant descent.

The present study extends the literature on acculturation focusing on teachers. We will test the conceptual model shown in Figure 1, including the proportion of students of immigrant descent as a covariate. We expect the following relations between our substantive variables:

*Hypothesis 1*: Teachers perceiving a school climate more strongly reflecting norms of *equality and inclusion* report a higher intercultural self-efficacy.

*Hypothesis 2*: Teachers perceiving a school climate more strongly reflecting norms of *cultural pluralism* report a higher intercultural self-efficacy.

*Hypothesis 3*: The positive effect of a perceived school climate of *equality and inclusion* on intercultural self-efficacy is mediated through a higher expectation for cultural adoption.

*Hypothesis 4*: The positive effect of a perceived school climate of *cultural pluralism* on intercultural self-efficacy is mediated through a higher support of cultural maintenance.

## Materials and methods

### Participants and procedure

The study used a cross-sectional design and was part of a larger study conducted in 2011 on the cultural diversity climate and acculturation in schools. The study was authorized by the Ministry of Education in the Federal State of Baden-Württemberg, Germany. A total of 207 teachers were recruited in 22 multiethnic secondary schools representing the three main secondary school tracks (streams) in the local school system: Seven low vocational track schools (*Hauptschule*), 10 medium vocational track schools (*Realschule*), and five high academic track schools (*Gymnasium*). Teachers had an average age of 41 years (*M*_age_ = 40.8; *SD*_age_ = 11.8) and were in service for an average of 13 years (*SD* = 0.67; range 1–32 years). About two thirds of the participants (73%) were female and 27 teachers (14%) reported to be of immigrant descent, 13 of whom were first-generation immigrants, mainly from Eastern Europe. In contrast to the teachers, the majority (69%) of the students at the sampled schools was of immigrant descent.

Within the schools, the project targeted students in 5th and 6th grade classrooms as well as the corresponding homeroom teachers and two teachers of the main subjects per classroom. Teachers were asked to complete the survey parallel to the students’ survey in their class where possible or received the survey with a written invitation and a small gift (chocolate) in their mailbox in the school office. Participation was voluntary and anonymous. Four in five questionnaires were returned and between three and 12 teachers per school participated in the survey (*M =* 8.45). Completed surveys were partly directly collected by the research team and partly in an envelope in the school office that was mailed back to the project leader later.

### Measures

Where possible, we chose established measures that were considered suitable for the use in Germany and with teachers. Where no suitable or adaptable measure was found, we adapted or developed our own measures based on the literature and exploratory interviews with four teachers (three female, two of immigrant descent) and one school principal (male, non-immigrant descent). Measures not originally available in German were translated using a translation/back-translation method. All measures applied 5-point Likert scales ranging from 1 (*do not agree at all*) to 5 (*fully agree*).

#### Diversity climate—equality and inclusion

The perceived *equality and inclusion* climate was assessed by adapted items from the School Interracial Climate Scale (Green et al., 1988; Molina and Wittig, 2006), mirroring parallel items for students (Schachner et al., 2016; Civitillo et al., 2017). The scale comprised three subscales: (a) perceived equal treatment and non-discrimination (seven items; e.g., ‘The teachers at this school are equally friendly to students with and without immigrant background.’), (b) support for contact and cooperation (four items; e.g., ‘In the composition of work groups, the teachers at this school make sure that they are mixed in terms of the cultural background of the students.’), (c) strengthening class community (four items; e.g., ‘The teachers at this school often organize joint activities to strengthen the classroom community.’).

#### Diversity climate—cultural pluralism

Perceived *cultural pluralism* climate was assessed using a combination of three subscales, which were newly devised for the purpose of the current study (also corresponding to student measures by Schachner et al., 2016): (a) dealing with cultural diversity constructively (12 items, e.g., ‘The teachers at this school try hard to boost the self-esteem of students of immigrant background.’), (b) appreciating cultural diversity (five items, e.g., ‘Most teachers at this school perceive it as enrichment for class when different traditions and ideas from students’ countries of origin are brought up and discussed.’), and (c) multicultural curriculum (five items, e.g., ‘The cultural background of immigrant students is regularly incorporated in class.’).

#### Acculturation expectations

Items measuring acculturation expectations were adapted from Arends-Tóth and van de Vijver (2006), with subscales measuring support for maintenance of the heritage culture and expectation for adoption of the mainstream culture, each containing three items. Exemplary items are ‘At school children of immigrant descent should be allowed to adhere to the values and norms of their country of origin’, for support for maintenance of the heritage culture and ‘At school children of immigrant background should comply with German values and norms’ for adoption of the mainstream culture.

#### Intercultural self-efficacy

Intercultural self-efficacy was measured with nine items, which had been developed for the purpose of this study (e.g., ‘I can adequately respond to students with different abilities and cultural preconditions.’).

#### Share of students of immigrant descent

We included the classroom proportion of students of immigrant descent as important school-level covariate of acculturation. The share of students of immigrant descent (*M* = 0.63; *SD* = 0.19*; range* = 0.24–0.94) was computed on the basis of the demographic information provided by 5th and 6th grade students at the respective schools who had participated in the corresponding student survey. Scores closer to 0 indicated a low share, whereas scores closer to 1 indicated a higher share of students of immigrant descent in the school.

### Analytic approach

We conducted several steps of analysis. First, we performed preliminary analyses on our measures, by conducting an Exploratory Factor Analysis (EFA), followed by Confirmatory Factor Analysis (CFA) to verify the factor structure of the scales. To judge model fit in the CFA, multiple indices were used, with comparative fit index (CFI) ≥0.90, Root Mean Square Error of Approximation (RMSEA) and Standardized Root Mean square Residual (SRMR) ≤0.08 indicating acceptable fit (Marsh et al., 2004), and CFI ≥0.95 and RMSEA and SRMR ≤0.06 indicating good fit (Hu and Bentler, 1999). In case of misfit, we examined the modification indices to see if the fit could be improved by omitting items that were expected to change the parameter by 0.2 (Brown, 2015). The Maximum Likelihood Robust (MLR) estimator was selected accounting for complete and incomplete data.

Hypotheses were tested using path analysis with a factor score regression approach. Factor scores were computed for all the latent constructs and saved for the main analysis. In case of the two climate dimensions, *equality and inclusion*, and *cultural pluralism*, we worked with a second-order factor summarizing the three subscales for each dimension instead of sum scores. This multistage approach is recommended when performing path analysis with small sample sizes (Rosseel, 2020). The hypothesized path model examined the direct and indirect associations among perceived *equality and inclusion* climate, perceived *cultural pluralism* climate, acculturation attitudes (i.e., support for the maintenance of the heritage culture, and expectation for adoption of the mainstream culture), and teacher intercultural self-efficacy, with proportion of students of immigrant descent in school as control. Model fit was evaluated considering similar fit indices as in CFAs. To estimate direct and indirect effects among pathways, we used bootstrapping procedure with 1,000 resamples. EFA, CFA and path analysis were conducted using JASP software (Version 0.14.1, JASP Team, 2020) and the *lavaan* package in *R* (4.0.2); (Rosseel, 2012).

## Results

### Preliminary analyses

Prior to analysis, patterns of missing values were examined. Twenty-one cases were excluded due to missing values on more than 20% of responses, leaving 186 cases for the final analyses. Missing values for the remaining participants were accounted for using the expectation–maximization-procedure in SPSS.

For *equality and inclusion*, we had theoretically assumed three subscales. These also emerged in the EFA, but we excluded six items because of low factor loadings (<0.40) or cross-loadings on other subscales. The CFA indicated an acceptable model fit on the remaining items, *χ*^2^(87) = 147.66, CFI = 0.920, TLI = 0.911, SRMR = 0.067, RMSEA = [0.58, 0.47]. The final subscale of perceived equal treatment and non-discrimination consisted of seven items (*α* = 0.77), the final subscale of support for contact and cooperation (*α* = 0.78) and the final subscale of strengthening class community (*α* = 0.67) consisted of four items each. For *cultural pluralism*, the expected three-factor structure emerged in EFA and was confirmed in CFA. The CFA indicated an acceptable model fit, *χ*^2^(227) = 366.77, CFI = 0.920, TLI = 0.911, SRMR = 0.067, RMSEA = [0.058, 0.47]. The subscales yielded good reliabilities for dealing with cultural diversity constructively (*α* = 0.89), appreciating cultural diversity (*α* = 0.76) and multicultural curriculum (*α* = 0.78).

Regarding the acculturation expectations, the three items measuring support for maintenance of the heritage culture were unifactorial in EFA and the subscale achieved a good reliability (*α* = 0.71). For the subscale measuring the expectation for cultural adoption, we excluded one item (“Children of immigrant background should generally only speak German on school grounds, even outside the classroom”) because it did not load on to the same factor and reduced the reliability of the scale. Many schools in Germany have formal rules that the use of heritage languages other than German is not allowed at school, which may be the reason why this item is less suitable to capture variations in teachers’ acculturation attitudes. The subscale expectation for adoption of the mainstream culture therefore contained two items. The most appropriate reliability statistic for a two-item scale is the Spearman–Brown coefficient (Eisinga et al., 2013), which in our case (*r* = 0.518, *p* < 0.001) indicated that there was a strong positive relationship between the two items (Dancey and Reidy, 2004). Due to the small number of items measuring acculturation attitudes, CFA were not conducted on these scales.

Regarding teachers’ intercultural self-efficacy, nine items were theoretically assumed unifactorial, but after conducting EFA, two factors emerged. This result is consistent with previous research by Civitillo et al. (2016) using a two factor scale to measure teachers self-efficacy in cultural diverse classrooms. We excluded one item from the scale measuring self-efficacy in culturally diverse classrooms because of cross-loadings with other items. CFA indicated a good model fit, *χ*^2^(19) = 52.13, CFI = 0.949, TLI = 0.925, SRMR = 0.057, RMSEA = [0.066, 0.129]. The subscale *teaching-related intercultural efficacy* (*α* = 0.86) consisted of three items, the subscale *efficacy in promoting positive intercultural relations* consisted of five items (*α* = 0.83). The final selection of items for the *Intercultural self-efficacy* scale, as well as the standardized factor loadings can be found in Table 1. Mean scores and standard deviations for our main study variables as well as correlations between the measures are presented in Table 2.

**Table 1:** Intercultural self-efficacy items, standardized factor loadings and reliabilities.

Dimension subscale	Item	Loading
Intercultural self-efficacy		
Teaching-related intercultural efficacy *α* = 0.86	In general I believe that I can cope with the challenges of a multicultural classroom.	0.80
	I can adapt my teaching to the cultural diversity of students.	0.89
	I can adequately respond to students with different abilities and cultural preconditions.	0.78
Efficacy in promoting positive intercultural relations *α* = 0.83	I can contribute to advance the relationship between students with and without migration background.	0.74
	I can take care that students with and without migrant background work together.	0.64
	I can raise awareness for cultural differences amongst the students.	0.73
	I can contribute to greater mutual understanding between students from different cultural groups.	0.79
	I can contribute to reducing mutual prejudices between the students.	0.61

**Table 2:** Correlations between main study variables (*N* = 186).

	M	SD	1	2	3	4	5	6	7	8	9	10
**Equality and Inclusion**												
1. Equal treatment and non-discrimination	5.52	0.55	–									
2. Contact and cooperation	3.23	0.87	0.174^*^	–								
3. Strengthening class community	4.18	0.61	0.316^***^	0.305^***^	–							
**Cultural pluralism**												
4. Dealing with cultural diversity constructively	3.38	0.60	0.251^***^	0.464^***^	0.499^***^	–						
5. Appreciating cultural diversity	2.32	0.72	–0.432^***^	–0.281^***^	–0.328^***^	–0.468^***^	–					
6. Multicultural curriculum	3.42	0.67	0.07	0.375^***^	0.356^***^	0.619^***^	–0.364^***^	–				
**Acculturation attitudes**												
7. Acceptance of cultural maintenance	3.16	0.93	–0.037	0.051	0.078	0.071	–0.161^*^	0.055	–			
8. Expectation for cultural adoption	3.99	0.79	0.068	0.078	–0.035	0.083	0.043	0.075	0.014	–		
**Intercultural self-efficacy**												
9. Teaching-related intercultural efficacy	3.68	0.76	0.244^***^	0.179^*^	0.287^***^	0.291^***^	–0.381^***^	0.142	0.025	0.147^**^	–	
10. Efficacy in promoting positive intercultural relations	3.92	0.61	0.158^*^	0.296^***^	0.263^***^	0.344^***^	–0.381^***^	0.287^***^	0.143^*^	0.023	0.499^***^	–

*** *p* < 0.001;

** *p* < 0.01;

* *p* < 0.05;

† *p* < 0.10.

### Path analysis

**Figure 2:**
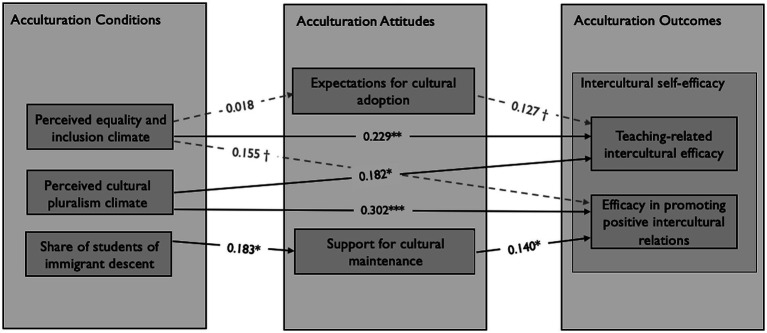
Final model with standardized coefficients.

The overall goodness of fit of the hypothesized model was good, χ^2^(18) = 163.06, CFI = 1.000, TLI = 1.105, SRMR = 0.008, RMSEA = [0.000, 0.037]. Overall, we could explain 15.7% of the variance in *teaching-related intercultural efficacy* and 20.5% of the variance in *efficacy in promoting positive intercultural relations*. The path model indicates that a perceived climate of *equality and inclusion* directly and positively predicts *teaching-related intercultural efficacy* (Hypothesis 1), whereas a perceived climate of *cultural pluralism* directly and positively predicts the *efficacy in promoting positive intercultural relations* (Hypothesis 2). There were no significant associations between either facet of the perceived diversity climate and acculturation expectations. Yet, a higher proportion of students of immigrant descent predicted a stronger support for cultural maintenance. Looking at the effects of acculturation attitudes on intercultural self-efficacy, only teachers’ support of cultural maintenance is associated with *teaching-related intercultural efficacy*. No significant mediation effects of acculturation attitudes were found in the associations between climate and facets of intercultural self-efficacy (Hypotheses 3 and 4). The results of our model are presented in Figure 2, standardized regression coefficients are reported^2^.

## Discussion

Teacher self-efficacy has been researched extensively in recent decades, but little attention has been paid to teacher self-efficacy in culturally diverse classrooms. Because many teachers feel unprepared and overwhelmed when dealing with cultural diversity, there is a need to understand how conditions at school can be more favorable for teachers’ intercultural self-efficacy. Our study is the first to apply an acculturation framework to teachers, who mainly represent the mainstream culture, while extending past work that focused on students of immigrant descent (Schachner et al., 2016). Thereby, we examined how teachers’ acculturation attitudes could potentially mediate the effects of the perceived cultural diversity climate on intercultural self-efficacy in culturally diverse classrooms. In doing so, we address the call to examine school (e.g., multicultural education; norms about diversity) and teacher characteristics (e.g., teachers attitudes) that could help us to understand when and why teachers feel more or less self-efficacious in relation to students of immigrant descent (Geerlings et al., 2018).

### School diversity climate and intercultural self-efficacy

Our findings are consistent with previous research showing associations between a positive school climate and higher (intercultural) self-efficacy amongst teachers (Gutentag et al., 2018; Schwarzenthal et al., 2022). Higher perceptions of an *equality and inclusion* climate were associated with higher *teaching-related intercultural efficacy*, meaning that teachers were more confident in coping with the challenges of a multicultural classroom and adapting their teaching to the cultural diversity of their students, as well as adequately responding to students with different abilities and cultural preconditions (Hypothesis 1). However, the lack of a positive association between perceived norms of *equality and inclusion* and the *efficacy of promoting positive intercultural relations* suggests that this approach does not fully address dealing adequately with cultural diverse classrooms. If teachers strongly emphasize equality, and follow a principle of neutrality to reduce the fear of discrimination, it may lead into a colorblind approach (Hachfeld et al., 2015; Schachner et al., 2016; Civitillo et al., 2017), which does not acknowledge the presence of discrimination and inequality and therefore may not be a suitable condition to address these issues with students and promote more positive intercultural relations. Indeed, colorblindness has been associated with lower awareness of cultural diversity, and lower *teaching-related intercultural efficacy* (Wang et al., 2014; Cadenas et al., 2021). Thus, our findings suggest that perceived norms of *equality and inclusion* are not sufficient to meet the needs of a multicultural classroom and should be accompanied by norms of *cultural pluralism* (Civitillo et al., 2017).

Teachers emphasizing *cultural pluralism* appear to have not only higher *teaching-related intercultural efficacy*, but also higher *efficacy regarding the promotion of positive intercultural relations*, i.e., they are more confident in advancing relationships between students of different cultural backgrounds, contributing to greater mutual understanding among students from different cultural groups, and helping to reduce mutual prejudices between students (Hypothesis 2). Teachers can contribute to students’ interethnic relationships not only through their cultural attitudes (Vezzali et al., 2012) but also through their own interpersonal relationships they build with their students (Thijs and Verkuyten, 2014). Thus, teachers can promote a positive cultural diversity climate by acting as role models and exemplifying norms of *cultural pluralism*: Positive relationships between teachers and students who belong to a different cultural group than the teachers can also improve students’ cultural attitudes in the classroom. However, research suggest that teachers have fewer positive relationships with students from other cultural groups than with students from their own cultural group (Thijs et al., 2012; Baysu et al., 2021), which in turn may negatively impact students’ evaluation of cultural differences in the classroom (Thijs and Verkuyten, 2014).

### Teachers’ acculturation expectations and intercultural self-efficacy

The findings we provide in this study give insights into teachers’ acculturation expectations and associations with teachers’ intercultural self-efficacy. Overall, we find only partial support for associations between teachers’ acculturation expectations and intercultural self-efficacy. Specifically, teachers with higher support for cultural maintenance feel more efficacious regarding culturally diverse students and promoting positive relationships amongst these. The benefits of appreciating diverse cultural backgrounds amongst students extends previous research suggesting similar positive effects, such as the appreciation of students’ heritage culture being associated with an increased motivation of the learners (Vedder and van Geel, 2012). Moreover, a higher support for cultural maintenance has been linked with higher diagnostic skills in assessing student characteristics, classroom social dynamics, and teachers’ classroom management (Makarova and Herzog, 2013).

Contrary to our established hypotheses (Hypothesis 3 and 4), and previous research on students’ acculturation (Schachner et al., 2016), we did not find that teachers’ acculturations expectations mediate the associations between school cultural diversity climate and intercultural self-efficacy. In contrast to the acculturation framework of students, which refers to one’s own acculturation orientations, the acculturation framework we applied in our current study to teachers (mainly representing mainstream society), refers to teachers’ expectations regarding the acculturation of students of immigrant descent. Our findings suggest that perceptions of school climate may be more salient than acculturations expectations, at least with regard to intercultural self-efficacy. These findings may also stem from the psychometric properties of the teacher acculturations expectations. For example, the scale assessing expectations for adoption of the mainstream culture only contains two items. Hence, future research is needed to further examine teachers’ acculturation expectations by using scales that are psychometrically more robust.

Our findings support previous research that teachers are more likely to accept cultural adoption compared to cultural maintenance (Makarova and Herzog, 2013). A simple explanation could be that teachers hope to socialize students of immigrant descent into the mainstream culture and prepare them for their (professional) future. Indeed, monocultural and monolingual norms are often dominant in schools (Moffitt et al., 2019). However, in everyday school life, if the expectation for cultural adoption is not complemented by the support for cultural maintenance, students of immigrant descent may feel that their cultural background is not sufficiently valued or even denigrated (Van Acker et al., 2014; Haenni Hoti et al., 2019). Teachers should be aware of the impact acculturation attitudes may have on students’ acculturation, academic achievement, and the teacher-student-relationship (Foster, 2008; Vedder and van Geel, 2012). But this is easier said than done because teachers might have unconscious biases which may have negative consequences for their expectations and behaviors towards students of immigrant descent (van den Bergh et al., 2010). Thus, our findings support the need for more attention to teacher education providing students in teacher education degrees opportunities to critically reflect on, examine, and discuss their own acculturation attitudes, but also their biases, stereotypes, and prejudices.

Finally, research indicated that effects of school composition (e.g., ethnic composition) has been associated with a wide range of teacher expectations (for a review see Brault et al., 2014). Our findings suggest that teachers in schools with a higher percentage of students of immigrant descent are more supportive of cultural maintenance, suggesting that greater diversity and intergroup contact is associated with more inclusive attitudes in schools. This is in line with previous findings and the contact hypothesis (Allport et al., 1954), which states that more frequent opportunities and higher quality intercultural contact can reduce prejudice. For instance, Berry et al. (2022) indicated that more intercultural contact is associated with more mutual acceptance and predicts a higher preference for integration.

### Limitation and future research

The present study should be considered in the light of some limitations. First, the data (perceived school climate, teacher attitudes, intercultural self-efficacy) are based on self-reports, i.e., they are sensitive to response tendencies and socially desirable responses (Paulhus, 2017; Kreitchmann et al., 2019). To obtain more reliable and robust data on school climate, future research could collect measures from multiple informants, such as student and teacher perceptions (Grazia and Molinari, 2021).

Second, we did not conduct multilevel analyses because of the small number of teachers in some schools (range from 2 to 14) and the small number of schools. Teachers share the same environment and may have similar perceptions of the school climate. Thus, multilevel analysis is crucial when observations are interdependent because it distinguishes contextual influences from individual influences (Mitchell et al., 2010).

Third, we measured public acculturation attitudes by phrasing our items specifically referring to the school context, which is considered part of the public domain, but some authors argue that norms and values are internalized and therefore private, even if measured in the school context (Arends-Tóth and van de Vijver, 2004; Kunst et al., 2012; Haugen and Kunst, 2017). Additionally, our datasets were cross-sectional, which makes it impossible to draw conclusions about causality. Kunst (2021) argues that acculturation is a causal and temporal phenomenon, which requires a longitudinal design to confirm the stability of the reported findings and draw conclusions about causality.

Forth, intercultural self-efficacy is based on self-reports, and does not necessarily reflect teachers’ classroom behaviors or students’ perceptions (Tschannen-Moran and Hoy, 2007). To obtain more reliable information about teachers’ intercultural self-efficacy and ultimately their interculturally competent behavior in the classroom, future research should couple self-reported intercultural self-efficacy with alternative measurement methods, e.g., situational judgment tests applied to teachers, interviews, or thinking-aloud procedures (Schwarzenthal et al., 2019b; Khukhlaev et al., 2021).

## Conclusion and implications

Despite the above-mentioned limitations, our study provides an insight into the importance of the acculturation context in schools (perceived cultural diversity climate and proportion of students of immigrant descent) and acculturation expectations on teachers’ intercultural self-efficacy. This seems particularly relevant for culturally diverse classrooms, as higher levels of intercultural self-efficacy among teachers buffer diversity-related stress (Gao and Mager, 2011; Ahsan et al., 2013) and increase the readiness to work in culturally diverse schools (Hachfeld et al., 2015). Our findings also contribute to the discussion on the education and preparation of teachers for cultural diversity and have several practical implications.

As both a perceived climate of *equality and inclusion* and a perceived climate of *cultural pluralism* were associated with teachers’ intercultural self-efficacy, implementing programs in schools fostering a school climate that encourages contact and collaboration but also celebrates cultural diversity is of high importance. However, schools should go beyond merely promoting *equality and inclusion* while developing a climate that welcomes *cultural pluralism*. Therefore, the appreciation of linguistic diversity and diverse backgrounds of students and their families can help fostering relationships with children and their families. Moreover, school leadership practices have a crucial role in constantly preparing culturally responsive teachers, providing culturally responsive school environments, and increasing teachers’ intercultural self-efficacy (Duyar et al., 2013; Khalifa et al., 2016).

Overall, the findings of this study provided useful insights into teachers’ perceptions of the school cultural diversity climate, as well as their acculturation expectations in relation to intercultural self-efficacy. Results suggest that efforts are needed to change deficit-oriented perspectives of teachers, so that diversity is valued as a resource for development and learning, not only for students but also for teachers (Vedder et al., 2006). Specifically entailing different opportunities for experiential learning (e.g., courses, mentoring, observation visits) are highly relevant for changing teachers’ beliefs about cultural diversity (Civitillo et al., 2018).

## Data availability statement

The raw data supporting the conclusions of this article will be made available by the authors, without undue reservation.

## Ethics statement

Ethical review and approval was not required for the study on human participants in accordance with the local legislation and institutional requirements. The patients/participants provided their written informed consent to participate in this study.

## Author contributions

JU: wrote the whole draft, participated in the design and the statistical analyses, MS: participated in the data collection, the research design, revised the manuscript and ran some first analyses on this research, SC: performed the statistical analyses and revised and reviewed the manuscript. PN: was involved in the original conceptualization of the study and reviewed the manuscript. All authors read and approved the final manuscript and are accountable for all aspects of the work in ensuring that questions related to the accuracy or integrity of any part of the work are appropriately investigated and resolved.

## Funding

The data collected are part of a larger study, which was funded by the federal program “ProExzellenz” of the Free State of Thuringia, which also provided a scholarship for MKS at the Graduate School of Human Behavior in Social and Economic Change at Friedrich Schiller University Jena.

## Conflict of interest

The authors declare that the research was conducted in the absence of any commercial or financial relationships that could be construed as a potential conflict of interest.

## Publisher’s note

All claims expressed in this article are solely those of the authors and do not necessarily represent those of their affiliated organizations, or those of the publisher, the editors and the reviewers. Any product that may be evaluated in this article, or claim that may be made by its manufacturer, is not guaranteed or endorsed by the publisher.

## Footnotes

^1^In Germany the public debate is often about culture, but culture is used in a racialized way and often implies ethnicity.

^2^As teacher data are nested within schools, we also ran the same path model again with robust standard errors to accommodate for the nested structure of the data. The results were very similar to the model reported, with all significant paths remaining significant.
